# The Relationship Between Maternal Periodontal Status of and Preterm and Low Birth Weight Infants in Iran: A Case Control Study

**DOI:** 10.5539/gjhs.v8n5p184

**Published:** 2015-09-28

**Authors:** Mohammad Reza Karimi, Jalaleddin H Hamissi, Simin Rafieyan Naeini, Mojgan Karimi

**Affiliations:** 1 Assistant Professor in periodontics, College of dentistry, Azad University, Teheran Branch, Teheran, Iran; 2 Associate Professor in periodontics & Preventive Dentistry, College of Dentistry, Qazvin University Medical Sciences, Qazvin, Iran; 3 Dentist, Iran

**Keywords:** low birth weight, preterm birth, periodontal disease

## Abstract

**Aim and Background:**

It has been suggested that periodontitis is associated with systemic alterations such as adverse pregnancy outcomes. However, some conflicting results have been reported. This study was conducted to determine the association between periodontitis and preterm birth (PTB), low birth weight (LBW) to obtain information which is necessary for the planning of preventive programs of periodontal disease for pregnant women in this area.

**Materials & Methods:**

This case-control study was performed on 264 mothers. The index used to determine oral hygiene and periodontal diseases is Community Periodontal Index Treatment Needs (CPITN).

**Results:**

The mothers in the sample group with single delivery delivered 8 times low birth weight infants more than the mothers in the control group with single delivery. And also the mothers in the sample group with multiple deliveries; delivered 10 times low birth weight infants and 8 times premature infant more than the mothers in the control group.

**Conclusion:**

More studies should be carried out in through preventing and treating periodontal diseases, expenses incurred due to preterm labor and low birth weight decrease and the society will witness fewer mental problems suffered by such children as they grow up. So we can emphasize the importance of periodontal care in prenatal health programs. And we may suggest that a special program of periodontal disease prevention for pregnant women is very necessary.

## 1. Introduction

Preterm infants are born prior to completion of 37 weeks of gestation (Honest et al., 2009; Epstein, Goldenberg, Hauth, & Andrews, 2000). An estimated 11% of pregnancies end in preterm birth (PB), (Goldenberg, Culhane, Iams, & Romero, 2008), and this rate appears to be on the rise in several developed countries, despite significant advances in obstetric medicine and improvements in prenatal care utilization. Of particular interest are the very preterm infants, born prior to 32 gestational weeks, the majority of which require neonatal intensive care due to their increased perinatal mortality, primarily due to impaired lung development and function (Beck et al., 2010; Manau, Echeverria, Agueda, Guerrero, & Echeverria, 2008).

Pregnancy is considered as one of complex physical and physiological changes that have significant impact on almost every organ system of the body. In the oral cavity, various pathologies have been reported among pregnant women (J. Hamissi, H. Hamissi, & Tabatabaei, 2012).

Periodontal disease is known as one of the most common chronic diseases with infectious origin in humans (Papapanou, 1996; Albandar & Rams, 2000; Offenbacher et al., 2001). It causes loss of the periodontal support of the teeth and it might lead to tooth loss (Albandar & Rams, 2002).

During pregnancy, 4 major hormones including Estrogen, Progesterone, HCG and HCS are produced in large amounts. Most of the time, increase of these hormones may bring some changes in gingiva. Results of clinical studies prove that 30% to 100% of pregnant women suffer from gingivitis (Folkers, Weine, & Wissman, 1992). Currently, periodontal disease is considered a risk factor for many systemic diseases such as preterm labor, or low birth weight (Sommerfelt, Troland, Ellertsen, & Markestad, 1996; Leitich, Bodner-Adler, Brunbauer, Kaider, Egarter, & Husslein, 2003; Lopez, Da Silva, Ipinza, Gutiérrez, 2005; Khader & Ta’ani, 2005). Perinatal morbidity and mortality is one of major cause of preterm birth and it has been increasing worldwide, reaching till 12% in the USA (Goldenberg, Culhane, Iams, & Romero, 2008).

Most of these studies were conducted in developed countries in spite of the fact that the vast majority of low birth weight babies are born in developing countries. To our knowledge, this association was not explored among Iranian women. The majority of women in Iran is of similar ethnic background, nonsmokers, nonalcoholic drinkers, and has only one sexual partner all through their life. Such characteristics were found to be associated with PLBW. Therefore, this study was conducted to assess the association between the severity and extent of maternal periodontal disease and PLBW delivery among women in the Qazvin. This study was carried out to investigate the impact of mother’s periodontal infections on the condition of the neonatal.

## 2. Method and Materials

This case–control study was conducted among women who gave birth at Kosar Teaching Hospital in the Qazvin, Iran over a period of 6 months. The study was approved by the Research Committee of the Qazvin University of Medical Sciences.

Four hundred six pregnant mothers were included. Those who had a history of spontaneous abortion, used antibiotics within the last three months of pregnancy and those afflicted with diabetes or hypertension and those addicted to cigarettes were excluded from the samples. The remained patient included 264 pregnant mothers. The index used to assess periodontal health was CPITN (Ainamo, Barmes, Beagrie, Cutress, Martin, & Sardo-Infirri, 1982).

The same examiner collected all data based on three criteria including examination, observation and questionnaire from all participants. Mothers were divided into two groups, those found in need of treatment (case group) and those who were healthy (control group). Each of these two groups were further divided into two subgroups based on gravidity, hence those who were giving birth to their first child were put in the same group and others were put in another group (each group contained 64 subjects). The group members were of the same age group and there was no significant difference among them regarding their education, job, and domicile. Mothers’ excess weights were the same in all groups. Height at birth was normal in all newborns both in case and in control groups and there was no significant difference in the circumferences of their heads. Pregnant women of both case and control groups were examined to find out whether they were in need of periodontal treatment (TN). None of the members of the multiparity and primiparity subgroups categorized under control group were in (no) need of periodontal treatment (TN=0). However, 58% of women in the primiparity subgroup categorized under case group proved to be in need of learning personal oral health to control dental plaque (TN=1) and 39% were in need of learning oral health principles and they also needed instructions to be provided by experts on methods of removing plaque and abolishing factors that retain plaque (TN=2). Moreover, 3% of these women required more complicated treatments such as oral surgeries (TN=3).

As to CPITN index, 58% of women included in the primiparity subgroup categorized under case group, were of code 1, and 27% were of code 2, 12% were of code 3 and 3% were of code 4. In multiparity subgroup categorized under case group, 24% were of code 1, 52% were of code 2, 15% were of code 3 and 9% were of code 4.

### 2.1 Statistical Analysis

Data was entered using the Epi Info computer program after which it was transferred to the SPSS, version 15, and program for analysis. Univariate analyses were performed by use of Chi-Square test and T-test was used wherever appropriate.

## 3. Results

Nine percent of mothers had babies with weights less than 2500gr at birth (LBW). 21% of mothers included in primiparity subgroup categorized under case group had babies with low weight at birth which reveals a significant difference (Table 1).

**Table 1:** A comparison of newborns’ average weight at birth in the study groups

Category	Group	Mean± std	Analysis table
**Primiparity**	Control	3071.82±421.6	(t=3.106, p=0.002)
	Case	2815.76±520.6	

**Multiparity**	Control	3233.33±395.2	(t=4.880, p=0.001)
	Case	2818.79±565.7	

In multiparity subgroup/control group, 3% of mothers had babies with low weight at birth, while18% of mothers included in multiparity subgroup categorized under case group had babies with low weight at birth, and this reveals a significant difference.

**Figure 1:**
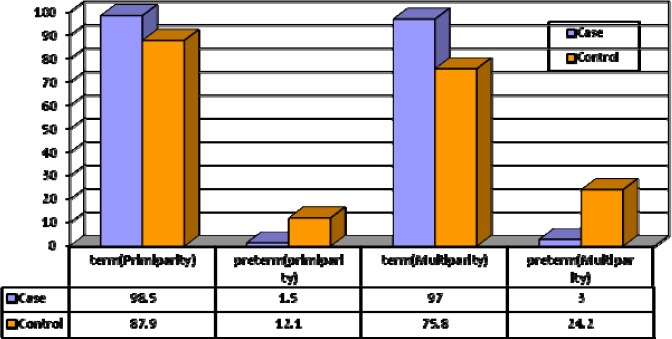
Distribution of newborns’ condition frequency in study groups

In primiparity subgroup categorized under control group, all mothers gave birth to babies after 37 weeks or more, however, in primiparity subgroup categorized under case group,12% of mothers had a pregnancy period of less than 37 and this shows a significant difference. In multiparity subgroup categorized under control group, 3% of women had pregnancy period of less than 32 weeks, however, in multiparity subgroup categorized under case group, 24% of mothers had pregnancy period of less than 37, which reveals a significant difference (Figure 1).

## 4. Discussion

The majority of the studies suggest that periodontal disease is associated with increased risk of various adverse pregnancy outcomes such as preterm birth and low birth weight. Infants are born preterm at less than 37 weeks’ gestational age (Slattery & Morrison, 2002).

According to the findings, we can conclude that the frequency of low birth weight in newborns of women who were giving birth to their first child and were afflicted with periodontal diseases were 2.3 times more than that of those newborns whose mothers were in periodontal health. This is somehow in disagreement with the findings of Offenbacher and those of Ketz because these authors believe that risk of preterm labor in mothers afflicted with periodontal diseases is six times more than that of healthy mothers (Hamissi et al., 2012). On the other hand, the frequency of low birth weight in those newborns whose mothers have experienced several labors and are afflicted with periodontal diseases is six times more than that of those newborns whose mothers are healthy. This is concordant with the findings of Afenbakher and Ketz. Moreover, findings of this study are concordant with those of Davenport et al (Davenport, Williams, Sterne, Sivapathasundram, Fearne, & Curtis, 1998) who proved that there is a relation between being afflicted with periodontal diseases and weight at birth.

As to the term of pregnancy, we can conclude that the frequency of preterm labor is 12 times more in women afflicted with periodontal diseases who are giving birth to their first child (pregnancy lasts for less than 37 weeks). Taking the study results into account, it can be concluded that term of pregnancy is significantly different between the primiparity subgroups categorized under case and control groups and this confirms the results achieved by Afenbakher, Ketz and Davenport. According to Toygar (2007), there is a relation between CPITN and low birth weight and as the CPITN increases in degree, the birth weight decreases and these concords with the results we achieved in this study (Toygar, Seydaoglu, Kurklu, Guzeldemir, & Arpak, 2007).

The present study demonstrated a 42.22% prevalence of CPITN score 3, corresponding to shallow pockets. Miyazaki et al. reported 31% of the pregnant women in a Japanese population had periodontal pockets of 4 or 5 mm, results similar to the data in the present work (Miyazaki et al., 1991).

Generally speaking, the more intensive the periodontal disease is, the more probable preterm labor and low birth weight will be and a reverse relationship exists between the average birth weight and the intensity of periodontal infections.

Ideally, women should begin their pregnancy without periodontal infections, and they should be educated and motivated to maintain a high level of oral hygiene prior to and throughout pregnancy. However, if a periodontal infection is diagnosed at any time during pregnancy, the treatment should be administered as soon as possible in order to reduce the risk of PT/LBW.

Health authorities should strengthen the implementation of community- based oral disease prevention and health promotion programmes. More importance must be given to oral health care center in family planning centers (J. Hamissi, Vaziri, & Davalloo, 2010).

## 5. Conclusion

More studies should be carried out in through preventing and treating periodontal diseases, expenses incurred due to preterm labor and low birth weight decrease and the society will witness fewer mental problems suffered by such children as they grow up. So we can emphasize the importance of periodontal care in prenatal health programs. Future research should focus on large-scale longitudinal studies, as well as interventional studies, to better prove a causal relationship and determine if periodontal treatment or prevention reduces the risk for adverse pregnancy outcomes.

**Recommendations**

- Additional epidemiological studies are needed including a larger number and wider spectrum of participants from different hospitals in different areas.
- Future studies in which the direct measurement of specific periodontal pathogens in the fetal environment and the measurement of the resulting inflammatory mediator levels are made would be helpful in either approving or disapproving the hypothesis.
- The use of clinical attachment level and gingival crevicular fluid as a clinical parameter.
- The use of the Decayed, Missing, or Filled Teeth Index (DMFT) as an extra measure to indicate the total number of decayed, missing, or filled teeth as a result of dental caries.
- Further studies are needed to address the question whether pre-term births can be reduced by treating the periodontal disease.
